# Comparison of Disease Patterns and Outcomes Between Non-Japanese and Japanese Patients at a Single Tertiary Emergency Care Center in Japan

**DOI:** 10.2188/jea.JE20200211

**Published:** 2022-02-05

**Authors:** Euma Ishii, Nobutoshi Nawa, Hiroki Matsui, Yasuhiro Otomo, Takeo Fujiwara

**Affiliations:** 1 Department of Global Health Promotion, Tokyo Medical and Dental University, Tokyo, Japan; 2 Department of Medical Education Research and Development, Tokyo Medical and Dental University, Tokyo, Japan; 3 Department of Acute Critical Care and Disaster Medicine, Tokyo Medical and Dental University, Tokyo, Japan

**Keywords:** non-national patient care, disease patterns, tertiary emergency center, Japan

## Abstract

**Background:**

Japan’s historically low immigration rate and monolingual culture makes it a particularly interesting setting for clarifying non-national medical care. Our study objective was to examine disease patterns and outcome differences between Japanese and non-Japanese patients in a rapidly globalizing nation.

**Methods:**

A secondary data analysis of 325 non-Japanese and 13,370 Japanese patients requiring tertiary care or intensive-care unit or high-care unit admission to the emergency department at the Tokyo Medical and Dental University medical hospital from 2010 through 2019 was conducted. Multivariable linear and logistic regressions models were applied to examine differences in percentage of diagnosis, mortality rates, and length of stay, stratified by Glasgow Coma Scale (GCS) scores to consider the impact of language barriers. Sex and age were adjusted.

**Results:**

Non-Japanese patients had more anaphylaxis, burns, and infectious disease, but less cardiovascular diagnoses prior to adjustment. After adjustment, there were significantly more anaphylaxis (adjusted odds ratio [aOR] 2.7; 95% confidence interval [CI], 1.7–4.4) and infectious disease diagnoses (aOR 2.2; 95% CI, 1.3–3.7), and marginally more burn diagnoses (aOR 2.3; 95% CI, 0.96–5.3) than Japanese patients. Regardless of GCS scores, there were no significant differences between non-Japanese and Japanese patient length of stay for anaphylaxis, burn, and infectious disease after covariate adjustment.

**Conclusion:**

There were more non-Japanese patients diagnosed with anaphylaxis, burns, and infectious disease, but no notable patient care differences for length of stay. Further prevention efforts are needed against anaphylaxis, burns, and infectious disease for non-Japanese tourists or residents.

## INTRODUCTION

Japan has seen a recent surge of non-Japanese tourists and residents due to globalization and revised immigration^1^ and work-status policies.^2^ In a decade, the annual number of tourists increased from 8.3 million in 2007 to 28.7 million in 2017,^3^ and the number of non-Japanese residents in Japan increased from 2 million in 2007 to 2.56 million in 2017.^4^ The government of Japan has also declared to increase the number of annual international visitors to 40 million by 2020 and 60 million by 2030.^5^

Despite the nation’s ambitious plans, the current status of non-Japanese patient care is understudied with limited quantitative research. There is ample research abroad, such as the United States,^6^ Canada,^7^ Korea,^8^ Germany,^9^ and Finland,^10^ that has focused on immigrant patient care outcomes; however, Japan has had significantly less immigrant patients than most nations,^11^ and therefore its healthcare system is nascent in non-Japanese patient care. Japan’s healthcare system is an excellent ecosystem to better understand issues related to non-national patient care and language and cultural barriers.^12^^–^^15^ Specifically for English language competency, Japan is third from last among the Organisation for Economic Co-operation and Development (OECD) nations.^16^ Japan also has had low immigration rates and has the lowest percentage of foreign-born residents in the world among developed nations,^17^^,^^18^ suggesting far fewer cases and thus less experience working with non-nationals.

The difficulty in obtaining healthcare access as an immigrant, especially for ambulatory and emergency care and regardless of having insurance coverage, is well understood.^19^ Previous studies by Kunii et al found that Japanese clinics prefer not to accept international patients due to communication difficulties, and the same can be said for international patients when visiting medical institutions.^20^ Language barriers can be particularly fatal in emergency medical settings where communication is essential to quickly and accurately check the medical history and patient’s state of consciousness.^21^ Furthermore, non-Japanese disease patterns in emergency care may differ from Japanese patients because of cultural differences. For these reasons, we find Japan to be an ideal setting to provide insights for improving non-national patient care outcomes, an imperative for an ever-globalizing world.

In this study, we used data from the Tokyo Medical and Dental University (TMDU) medical hospital’s Trauma and Acute Critical Care Center that provides tertiary emergency care.^22^ The aim of this study was to clarify the disease pattern and outcome differences between Japanese and non-Japanese patients at TMDU’s medical hospital to uncover the actual conditions of non-Japanese patient care in a tertiary emergency care setting in Japan. In addition, we hypothesized that we would observe less outcome differences between non-Japanese and Japanese patients for those with Glasgow Coma Scale (GCS) scores less than 13, which denotes unconsciousness,^23^ as communication difficulties would likely not be relevant. Thus, our study also aims to examine the outcome differences between Japanese and non-Japanese patients, stratified by the patient’s level of consciousness. By analyzing emergency department (ED) patient data, we hope our results will elucidate the differences in non-Japanese and Japanese patient care, to better host non-Japanese residents and tourists in Japan.

## METHODS

### Study design and setting

Our baseline data consisted of 19,420 Japanese and non-Japanese patients that either required tertiary care that were transported to the TMDU medical hospital’s Trauma and Acute Critical Care Center, or were admitted to the ED intensive-care unit (ICU) or high-care unit (HCU) between January 1, 2010 to December 31, 2019. TMDU’s medical hospital is located in Tokyo, the capital city of Japan, where the number of international tourists increased from 5.3 million in 2007 to 14.3 million in 2017,^3^ and the number of residents from 402,432 in 2007 to 555,063 in 2017.^24^ The hospital, juxtaposed to multiple tourist sites including the imperial palace, provides tertiary emergency care^22^ and currently admits one of the highest number of patients among university hospital EDs.^25^ TMDU’s medical hospital is not Japan Medical Service Accreditation for International Patients (JMIP) or Japan International Hospital (JIH) accredited. In general, the non-Japanese patient flow is identical to Japanese patients. When the non-Japanese patient cannot speak Japanese nor English, the patient tends to bring a family member or friend that can communicate with the healthcare professionals at TMDU. In rare cases where the non-Japanese patient does not have a family member or friend to translate, healthcare professionals use online translation tools to communicate.

### Selection of patients

**Figure 1.**
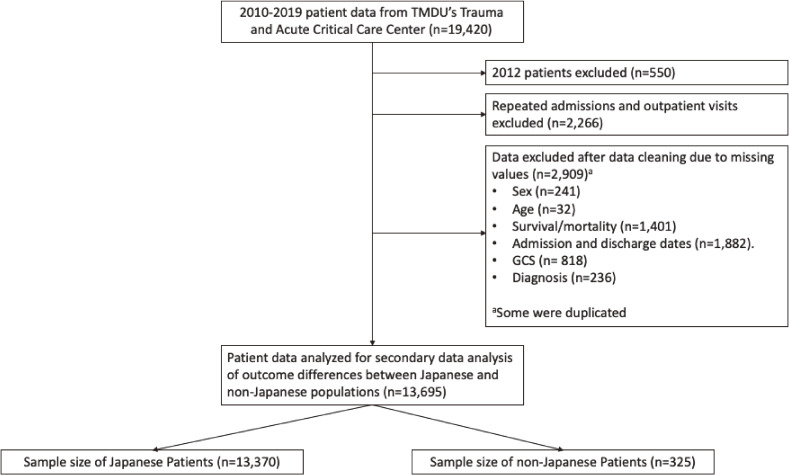
Flowchart for patient selection process

As shown in Figure 1, we first excluded all patients from 2012 (*n* = 550), as this year had a power outage in which only the first 6 months of data were recovered by manually entering data. We did not include any follow-up tertiary outpatient visits or admissions (*n* = 2,266) from the same patient and focused on the first-time tertiary outpatient visit or admission as repeated visits or admissions may distort the observed disease pattern and outcome of the patients. The sample for the analysis also excluded patients that had any missing values for the covariates of interest (*n* = 2,909, see Outcomes section below for full list). Further details for the number of missing cases for Japanese and non-Japanese patients for the covariates of interest are described in eTable 1. The non-Japanese population did not necessarily have a higher proportion of missing values in comparison to the Japanese population. After applying our exclusion criteria, a sample size of 13,695 consisting of 325 non-Japanese and 13,370 Japanese patients was used for the analysis. The research was approved by the TMDU’s Ethics Review Committee of the School of Medicine (IRB approval number: M2019-293).

### Measurements

#### Nationalities

Since it is not mandatory for clinicians at hospitals to query patients about their nationalities,^26^ the data did not include information concerning nationalities. Prior to anonymization, patient names were categorized into Japanese and non-Japanese, and then separated further into ethnicities. The rationale for name-based categorization includes the following: Specific Chinese characters (*kanji*) are not used in Japan or for Japanese names,^27^ therefore these names were included in the non-Japanese sample. Additionally, names only using *katakana*,^28^ another system of writing in Japan specifically for loanwords, were categorized as non-Japanese alongside alphabet-based names. All names were further separated by ethnicity; 1) East Asian (Chinese or Korean names written in *katakana* or k*anji*, are included in the last name), 2) South Asian, South East Asian, Middle East (names that are of Asian descent, but not East Asian), 3) Caucasian and Hispanic (western names for both first and last names and names with Spanish connotation), and 4) Unspecified (non-Japanese names with Japanese last names as this may be due to marriage). Further details are included in eTable 2. After anonymization, flags were placed using the aforementioned rationale to categorize Japanese and non-Japanese patients. Further, this dataset included clinical summaries permitting inference for disease onset and patient pathway to hospital.

#### Outcomes

Diagnosis, length of stay, and mortality were used as dependent variables. Our study was interested in clarifying the actual situation of non-Japanese patient care in Japan because non-Japanese patients have barriers to healthcare access, such as language or communication difficulties, leading to poorer access to health and potentially increased severity of illness.^20^^,^^21^ In this study we used frequency of disease, mortality, and length of stay to elucidate the actual situation of non-Japanese patient care in Japan. Since severity is part of the actual situation and the difference would be lost after adjustment, we chose not to adjust for severity. Specific diagnoses were initially documented by ED physicians upon patient visit (primary disease), which was then categorized based on the Eleventh Edition of the Principal Diagnosis Short List for Emergency Medicine which incorporates the World Health Organization’s (WHO) International Classification of Diseases^29^: cardiovascular disease; cardiopulmonary arrest on arrival (CPAOA); cardiopulmonary arrest (CPA, at hospital/after arrival); respiratory disease; gastrointestinal disease; neurological disease; traumatic injury; overdose (including toxins, poisons); endocrine disease; blood or immune system disease (hemo-immune); infectious disease; burns; anaphylaxis; self-harm (eg, suicide attempt); psychiatric disorders (mental, behavioral, or neurodevelopmental disorders); genitourinary (any disease characterized by pathological changes to the genitourinary system), and others (unclassified). Further details are included in eTable 3. Length of stay was defined as the entire length of hospital stay, determined by subtracting the discharge date (discharge from TMDU medical hospital including ED-ICU, ED-HCU, general ward, interhospital or intrahospital transfer, or ED outpatient care) from the hospital visit or admission date (ED outpatient visit date or admission to TMDU medical hospital including ED-ICU, ED-HCU, or general ward) and adding one. Specifically, our sample included cases requiring tertiary care with same day discharge after outpatient treatment alone, and transfers from the ED outpatient care to other TMDU hospital departments or to other hospitals. The length of stay for these patients was set at 1 day as ED care was completed within a day. Mortality rates were determined by survival and in-hospital mortality labels within the database. Further, for deeper understanding of the situation, we also reviewed anonymized clinical summaries.

#### Covariates and potential effect modifiers

Adjusted covariates were sex and age (Table 1). These covariates were selected based on prior studies assessing patient outcome differences between nationals and non-nationals in the United States^30^ and Europe.^31^ GCS scores were chosen for a potential effect modifier since we hypothesized that less outcome differences would be observed between non-Japanese and Japanese patients for those with low GCS scores (GCS <13), as communication barriers would not likely be relevant. This cutoff was based on previous literature denoting patients with GCS scores above 13 as conscious, and scores below 13 as patients with poorer levels of consciousness.^23^

**Table 1.** Demographics, population, length of stay, severity, mortality rate, and analysis of Japanese and non-Japanese patients

	Years		Non-Japanese (*N* = 325) *N* (%)	Japanese (*N* = 13,370) *N* (%)	*P*-value^a,b,c^
Male	2010–2019		194 (59.7%)	8,758 (65.5%)	**0.03**

Age	2010–2019	0–14	10 (3.1%)	273 (2.0%)	
		15–64	272 (83.7%)	6,694 (50.1%)	
		≥64	43 (13.2%)	6,403 (47.9%)	
		Mean (SD)	40.6 (19.0)	60.2 (21.1)	**<0.01**

Ethnicity	2010–2019	East Asian	189 (58.2%)		
		South Asian/ South East Asian/ Middle East	50 (15.4%)		
		Caucasian/ Hispanic	68 (20.9%)		
		Unspecified	18 (5.5%)		

Total population by year	2010		14 (4.3%)	1,198 (9.0%)	
	2011		25 (7.7%)	1,253 (9.4%)	
	2012		—	—	
	2013		28 (8.6%)	1,620 (12.1%)	
	2014		34 (10.5%)	1,614 (12.1%)	
	2015		37 (11.4%)	1,505 (11.3%)	
	2016		33 (10.2%)	1,539 (11.5%)	
	2017		47 (14.5%)	1,445 (10.8%)	
	2018		46 (14.2%)	1,493 (11.2%)	
	2019		61 (18.8%)	1,703 (12.7%)	

Glasgow Coma Scale	2010–2019	GCS ≥13	100 (30.8%)	4,025 (30.1%)	0.84
		GCS <13	225 (69.2%)	9,345 (69.9%)	

Mortality	2010–2019		27 (8.3%)	1,539 (11.5%)	0.09

Length of stay, days	2010–2019	Median (IQR)	2.0 (6.0)	3.0 (8.0)	**0.03**

GCS, Glasgow Coma Scale; IQR, interquartile range; SD, standard deviation.

^a^Categorical values are analyzed using Chi-Square test.

^b^Continuous values are analyzed using T-Test or Mann-Whitney U Test.

^c^*P*-values <0.05 are shown in bold.

Severity proportion based on the Glasgow Coma Scale.

TMDU’s emergency department from 2010–2019 (except 2012, *N* = 13,695).

### Analysis

Mean age and length of stay (in days) were compared using *t*-tests and Mann-Whitney U tests between non-Japanese and Japanese patients (Table 1). Percentage of males, GCS scores lower than 13 and death were compared using chi-squared tests between non-Japanese and Japanese patients (Table 1).

Disease diagnoses, mortality, and length of stay were compared between non-Japanese and Japanese by chi-square for disease diagnosis and mortality (and Fisher’s exact test), and Mann-Whitney U test for length of stay (Table 2). We then selected results with the greatest difference (*P* < 0.2) in percentage of diagnosis between non-Japanese and Japanese patients, stratified by GCS scores, and used logistic regressions to compare disease diagnoses (Table 3). Linear regressions were used to compare the length of stay between non-Japanese and Japanese patients (Table 4). Both models were adjusted for age and sex. Among the selected diagnoses (cardiovascular, anaphylaxis, burns, and infectious disease), since there was only 1 mortality (infectious disease) within the non-Japanese patient sample, we did not include the analysis for mortality using the selected diagnoses. All analyses were conducted by R Version 3.5.3 (R Foundation for Statistical Computing, Vienna, Austria).^32^ with complete data without missing values. Statistical significance was set at *P* < 0.05.

**Table 2.** Percentage of diagnosis, mortality rate, length of stay, and analysis of Japanese and non-Japanese patients

Diagnosis	Total			Mortality			Length of Stay (LOS)		
		
	Non-Japanese *n* (%)	Japanese *n* (%)	χ^2^ Test *P*-value	Non-Japanese *n* (%)	Japanese *n* (%)	Fisher Test *P*-value	Non-Japanese Median (IQR)	Japanese Median (IQR)	Mann-Whitney *P*-value
		
Injury	63 (19.4%)	2,892 (21.6%)	0.37	7 (2.2%)	111 (0.8%)	**0.01**	2.0 (3.5)	3.0 (6.0)	**0.03**
Gastrointestinal	56 (17.2%)	2,000 (15.0%)	0.29	0 (0.0%)	82 (0.6%)	0.17	5.0 (6.0)	6.0 (8.0)	0.25
Cerebrovascular	37 (11.4%)	1,423 (10.6%)	0.74	4 (1.2%)	142 (1.1%)	0.78	4.0 (12.0)	6.0 (12.0)	0.33
Cardiovascular	14 (4.3%)	1,097 (8.2%)	**0.01**	0 (0.0%)	96 (0.7%)	0.62	3.5 (5.0)	3.0 (11.0)	0.98
Overdose/Toxin	28 (8.6%)	965 (7.2%)	0.39	0 (0.0%)	11 (0.1%)	>0.99	2.0 (1.0)	2.0 (2.0)	0.24
CPAOA	17 (5.2%)	803 (6.0%)	0.64	13 (4.0%)	740 (5.5%)	**0.04**	2.0 (8.0)	1.0 (0.0)	**<0.01**
Neurological	14 (4.3%)	806 (6.0%)	0.24	0 (0.0%)	11 (0.1%)	>0.99	1.0 (1.0)	2.0 (3.0)	0.26
Pulmonary	16 (4.9%)	612 (4.6%)	0.87	1 (0.3%)	57 (0.4%)	>0.99	3.5 (5.5)	5.0 (8.0)	0.62
Infectious	17 (5.2%)	486 (3.6%)	0.17	1 (0.3%)	52 (0.4%)	>0.99	4.0 (5.0)	7.0 (11.0)	**0.03**
Endocrine	6 (1.8%)	286 (2.1%)	0.87	0 (0.0%)	13 (0.1%)	>0.99	5.0 (3.8)	4.0 (9.0)	0.55
CPA	5 (1.5%)	270 (2.0%)	0.68	1 (0.3%)	101 (0.8%)	0.65	4.0 (2.0)	4.0 (9.0)	0.69
Anaphylaxis	21 (6.5%)	176 (1.3%)	**<0.01**	0 (0.0%)	3 (0.02%)	>0.99	2.0 (0.0)	2.0 (1.0)	0.09
Burns	6 (1.8%)	76 (0.6%)	**<0.01**	0 (0.0%)	3 (0.02%)	0.99	1.5 (1.0)	3.0 (9.3)	0.11
Self-Harm	2 (0.6%)	66 (0.5%)	>0.99	0 (0.0%)	37 (0.3%)	0.20	4.5 (3.5)	1.0 (1.0)	0.46
Hemo-Immuno	1 (0.3%)	59 (0.4%)	>0.99	0 (0.0%)	5 (0.04%)	>0.99	2.0 (—)	6.0 (9.5)	0.28
Psychiatry	2 (0.6%)	76 (0.6%)	>0.99	0 (0.0%)	0 (0.0%)	—	1.0 (0.0)	2.0 (3.3)	0.17
Genitourinary	0 (0.0%)	25 (0.2%)	0.90	0 (0.0%)	0 (0.0%)	—	— (—)	6.0 (12.0)	—
Other	20 (6.2%)	1,252 (9.4%)	0.06	0 (0.0%)	75 (0.6%)	0.63	2.0 (4.5)	3.0 (7.0)	0.30

CPA, cardiopulmonary arrest; CPAOA, cardiopulmonary arrest on arrival; LOS, length of stay; SD, standard deviation.

Non-Japanese (*n* = 325) and Japanese patients (*n* = 13,370).

^*^P-values <0.05 are shown in bold.

“—” denotes *n* = 0 or 1 for either the Non-Japanese or Japanese sample, and thus cannot be computed.

TMDU’s emergency department from 2010–2019 (except 2012, *N* = 13,695).

**Table 3.** Multivariable analysis of the percentage of specific diagnoses for Japanese and non-Japanese patients

		Percentage			
		
Diagnosis	GCS Score	Non-Japanese *n* (%)	Japanese *n* (%)	aOR	95% CI
Cardiovascular		14 (4.3%)	1,097 (8.2%)	0.9	(0.5, 1.5)
	GCS ≥13	13 (4.0%)	859 (6.4%)	1.1	(0.6, 1.9)
	GCS <13	1 (0.3%)	238 (1.8%)	0.3	(0.04, 2.1)
Anaphylaxis		21 (6.5%)	176 (1.3%)	**2.7**	**(1.7, 4.4)**
	GCS ≥13	18 (5.5%)	167 (1.2%)	**2.4**	**(1.4, 4.1)**
	GCS <13	3 (0.9%)	9 (0.1%)	**9.2**	**(2.4, 35.6)**
Burns		6 (1.8%)	76 (0.6%)	2.3	(0.96, 5.3)
	GCS ≥13	6 (1.8%)	67 (0.5%)	**2.6**	**(1.1, 6.3)**
	GCS <13	0 (0.0%)	9 (0.1%)	—	—
Infectious		17 (5.2%)	486 (3.6%)	**2.2**	**(1.3, 3.7)**
	GCS ≥13	11 (3.4%)	360 (2.7%)	**2.0**	**(1.04, 3.7)**
	GCS <13	6 (1.8%)	126 (0.9%)	**3.1**	**(1.3, 7.4)**

aOR, adjusted odds ratio (referent is Japanese patients, adjusted for sex and age); GCS, Glasgow Coma Scale.

Non-Japanese patients (*n* = 325) and Japanese patients (*n* = 13,370).

Odds ratios adjusted for: sex and age.

Analyzed with multivariable regression adjusted for sex and age.

“—” denotes *n* = 0 or 1 for either the Non-Japanese or Japanese sample, and thus cannot be computed.

^*^P-values <0.05 are shown in bolds.

TMDU’s emergency department from 2010–2019 (except 2012, *N* = 13,695).

**Table 4.** Multivariable analysis of the length of stay of Japanese and non-Japanese patients with specific diagnoses

		Length of Stay				
Diagnosis	GCS Score	Non-Japanese Median (IQR)	Japanese Median (IQR)	Mann-Whitney *P*-value	Coeff	95% CI
					
Cardiovascular		3.5 (5.0)	3.0 (11.0)	0.98	−5.5	(−16.9, 5.9)
	GCS ≥13	3.0 (3.0)	3.0 (11.0)	0.70	−6.5	(−18.6, 5.6)
	GCS <13	22.0 (—)	3.0 (12.0)	0.25	10.1	(−29.8, 50.0)

Anaphylaxis		2.0 (0.0)	2.0 (1.0)	0.09	0.4	(−0.1, 0.9)
	GCS ≥13	2.0 (0.0)	2.0 (1.0)	0.08	0.4	(−0.1, 0.98)
	GCS <13	2.0 (0.5)	1.0 (1.0)	0.53	0.2	(−0.9, 1.4)

Burns		1.5 (1.0)	3.0 (9.3)	0.11	−5.5	(−19.5, 8.5)
	GCS ≥13	1.5 (1.0)	2.0 (9.5)	0.15	−6.0	(−20.5, 8.4)
	GCS <13	— (—)	7.0 (5.0)	—	—	—

Infectious		4.0 (5.0)	7.0 (11.0)	**0.03**	−8.5	(−17.7, 0.6)
	GCS ≥13	4.0 (5.0)	7.0 (11.0)	0.14	−8.9	(−20.2, 2.3)
	GCS <13	4.0 (2.3)	8.0 (13.0)	0.14	−9.0	(−25.2, 7.2)

CI, confidence interval; Coeff, coefficient; GCS, Glasgow Coma Scale; IQR, interquartile range; SD, standard deviation.

Coefficient adjusted for: sex and age.

Non-Japanese patients (*n* = 325) and Japanese patients (*n* = 13,370).

Analyzed with multivariable regression adjusted for sex and age.

“—” denotes *n* = 0 or 1 for either the Non-Japanese or Japanese sample, and thus cannot be computed.

^*^P-values <0.05 are shown in bold.

TMDU’s emergency department from 2010–2019 (except 2012, *N* = 13,695).

## RESULTS

Table 1 summarizes the comparison between a non-Japanese sample of 325 (2.4%) to a Japanese sample of 13,370 (97.6%) of characteristics (sex, age, ethnicity), population per year, the percentage of GCS scores less than 13, and mortality. Non-Japanese patients (mean 40.6; standard deviation [SD], 19.0 years) were significantly younger than Japanese patients (mean 60.2; SD, 21.1 years). The median length of stay was significantly lower in the non-Japanese sample (2.0; interquartile range, [IQR], 6.0 days) in comparison to the Japanese sample (3.0; IQR, 8.0 days).

Table 2 outlines the percentage of each diagnosed disease, mortality rate, and mean length of stays. Among non-Japanese patients, there were more anaphylaxis (6.5% vs 1.3%, *P* < 0.01), burn (1.8% vs 0.6%, *P* < 0.01), and infectious disease (5.2% vs 3.6%, *P* = 0.17) diagnoses and less cardiovascular (4.3% vs 8.2%, *P* = 0.01) diagnoses. Regarding death, there were significantly more injury-related deaths (2.2% vs 0.8%, *P* = 0.01), but significantly less CPAOA (4.0% vs 5.5%, *P* = 0.04) related deaths for non-Japanese patients. In addition, non-Japanese patients had significantly lower median length of stay for injury (2.0 vs 3.0 days, *P* = 0.03) and infectious disease (4.0 vs 7.0 days, *P* = 0.03) diagnoses, and significantly greater length of stay for CPAOA (2.0 vs 1.0 days, *P* < 0.01) diagnoses. Therefore, we focused on cardiovascular, anaphylaxis, burns, and infectious disease as these showed the greatest difference (*P* < 0.2) in percentage of diagnosis (Table 2). eTable 4 includes the age-stratified percentage of diagnosis and length of stay for non-Japanese and Japanese patients. For all four diagnoses, Japanese patients had a higher proportion of elderly patients than the non-Japanese sample (eTable 4).

According to the clinical summaries, all 21 non-Japanese anaphylaxis cases were food-induced, with 15 cases (71%) triggered by the Japanese traditional “soba” (buckwheat) noodles, fish, or shellfish. Furthermore, all of the non-Japanese anaphylaxis patients were tourists from abroad. In contrast, 106 Japanese patients with anaphylaxis were triggered by food (60%), and 53 cases (30%) were due to medication or treatment. Notably, for drug-induced anaphylaxis, 16 cases (30%) of 53 cases were due to dental prescriptions or treatment. All non-Japanese patients with burn diagnosis were outside of their homes during their incidents. For instance, there were many employees working in non-ideal conditions. For Japanese patients with burn diagnoses, 61 cases (80%) occurred at home, with a third of these cases occurring in the bath, and a third while cooking. Regarding non-Japanese infectious disease patients, no specific patterns were observed, whereas the Japanese sample had a high number of sepsis cases at 118 (24%). However, elderly non-Japanese patients with infectious disease tended to be residents with prior chronic illnesses that were diagnosed with infectious diseases.

Table 3 displays the odds ratio of disease diagnoses by a multivariable logistic regression adjusted for sex and age, stratified by GCS scores. There were significantly more anaphylaxis (adjusted odds ratio [aOR] 2.7; 95% confidence interval [CI], 1.7–4.4) and infectious disease (aOR 2.2; 95% CI, 1.3–3.7) cases for non-Japanese regardless of GCS scores. When stratified by GCS scores, there were significantly more non-Japanese anaphylaxis cases (GCS ≥13: aOR 2.4; 95% CI, 1.4–4.1, and GCS <13: aOR 9.2; 95% CI, 2.4–35.6) and infectious disease for both consciousness levels (GCS ≥13: aOR 2.0; 95% CI, 1.04–3.7, and GCS <13: aOR 3.1; 95% CI, 1.3–7.4) compared to Japanese cases. As for burns, there were marginally more non-Japanese patients after covariate adjustment (burns: aOR 2.3; 95% CI, 0.96–5.3, *P* = 0.092), and significantly more burn diagnoses in non-Japanese patients with GCS ≧13 (aOR 2.6; 95% CI, 1.1–6.3) in comparison to Japanese patients. Finally, for cardiovascular diagnoses, adjusting for sex and age, there were no significant differences between non-Japanese and Japanese patients regardless of GCS score stratification.

Table 4 summarizes the results for a multivariable analysis of the length of stay using a linear regression adjusted for sex and age stratified by GCS scores. After adjustment for sex and age, we found no significant differences between non-Japanese and Japanese patients for all four diagnoses.

eTable 5, eTable 6, and eTable 7 compare the percentage of diagnosis, mortality rate, and length of stay, respectively, between the specific ethnicities of non-Japanese patients with the Japanese patients. When compared with the Japanese patient sample, there were significantly larger percentages of Caucasian and Hispanic group and the East Asian group for anaphylaxis compared to Japanese patients, whereas the South/Southeast Asian and Middle Eastern group had significantly more percentages of burn and overdose diagnoses (eTable 5). We also observed in the clinical summaries that although the South/Southeast Asian and Middle Eastern patients had consumed various substances such as bleach, preservatives, and prescribed medications, none of the patients diagnosed with overdose had consumed alcohol nor opiates. We found significantly more deaths for East Asian patients with injury diagnoses than Japanese patients (eTable 6). According to the clinical summaries, the majority of East Asian patients diagnosed with injury were due to car accidents (30%) and injuries while under the influence of alcohol (22%). Furthermore, 18% of the patients who arrived with injuries from car accidents were reported to be intoxicated. After stratification by ethnicity, when diagnosed with CPAOA, the East Asian patients and the South/Southeast Asian and Middle Eastern patients had significantly longer lengths of stay in comparison to Japanese patients (eTable 7).

## DISCUSSION

In this study, we found there were more anaphylaxis, burn, and infectious disease diagnoses in non-Japanese patients when compared to Japanese patients. There were no differences between non-Japanese and Japanese patients for mortality rates or mean lengths of stay even after taking language barriers into account through stratification of GCS scores. As TMDU is a tertiary emergency setting, the patient cohort’s severity may be evenly distributed, which may have affected our outcomes even after taking age into account. This is also consistent with previous research conducted at a tertiary emergency care center, which found that there were no significant differences in age for patients that died and survived.^33^

In 2018, it was reported that 5% of international tourists visiting Japan suffered unexpected injuries or illnesses, of which 26% felt the need to receive professional care.^34^ Our results reflected much of the previous literature examining non-national patient samples. Regarding anaphylaxis, multiple studies including a research at a tertiary center in Qatar (where 85% of the population are expatriates) found that food-induced cases were the most common reason for onset.^35^ Buka et al also determined that there were significantly more non-national (in this case, South Asian) anaphylaxis cases than Caucasian patients in the United Kingdom.^36^ This was consistent with our results in eTable 5 where non-national groups (Caucasian and Hispanic and the East Asian group) had significantly more anaphylaxis diagnoses over the national group (Japanese). Considering the length of stay, Hasegawa et al conducted a multicenter study in the United States and found there were no significant differences in the length of stay between ethnicities diagnosed with anaphylaxis regardless of age or sex,^37^ which after adjustment was consistent with our findings.

To prevent anaphylaxis among non-Japanese, although many traditional Japanese-style restaurants focus on a particular dish or style of cooking and the predominant native population would know what was inside each dish, the rate of anaphylaxis may be higher for the non-Japanese population due to gaps in the information provided. While all consumable products sold in Japan list ingredients, and explicitly note potential allergens, the majority tend to only be written in Japanese. In addition, although major restaurants have begun to provide English or multilingual menus, it is not mandatory in Japan to provide allergen information in writing at restaurants,^38^ unlike Western countries such as the United Kingdom.^39^ Specifically, as we found the majority of non-Japanese anaphylaxis emergency care visits were due to “*soba*” (buckwheat) or fish and shellfish, it may be preferable to inquire whether visitors have allergies to these prior to taking their orders, and also document these allergens in the menus.

As for burns, previous studies^40^^–^^42^ also reported higher proportions of burn diagnoses in migrants and migrant workers. Children of migrant workers were found to be at significantly higher risk than those of nationals in Shanghai^40^ and a study of the British Columbia Professional Firefighters Burn Unit registry found that Asians had the highest occurrences of workplace injuries and a higher proportion of scald injuries compared to other ethnicities.^41^ The latter article suggested the need for language-specific instructions and workshops to prevent future injuries.^41^ A study conducted in the United Kingdom also found that over 40% of the total patient cohort presenting burns were Asian ethnic minorities, of which approximately 30% were caused by contact burns including hot iron use in the workplace.^42^ This was consistent with our findings, where South/Southeast Asian and Middle Eastern patients had significantly higher percentages for burn diagnoses. Further, Papp et al found that minority Asian patients had shorter length of hospital stays when compared to Caucasian patients diagnosed with burns prior to age and sex adjustment in Canada.^41^ A literature review of burn studies found significant correlations between patients with burns and their socio-economic status including; ethnicity (non-Caucasian), low income, larger families, single parents, illiteracy, low maternal education, unemployment, and substandard living conditions such as homelessness.^43^ Although the total number of burn patients in our study is small, as Japan expands its immigration laws to accept more international workers,^2^ it may be helpful to have written documentation for fire extinguishing, as well as mandatory emergency fire-extinguishing lectures conducted in English (or their native tongue) to prevent further accidents when hiring non-national workers.

Regarding infectious disease, a previous report in the United States established that infectious disease proportions were significantly higher for immigrants when compared to nationals^44^ as seen with our study. In New Zealand, ethnicity, age, or sex was found to not affect the length of stay for patients diagnosed with infectious disease,^45^ which was also consistent with our study. However, our results differed from a Norway Institute of Public Health study that reported significantly more immigrant HIV, tuberculosis, and hepatitis B cases over nationals.^46^ The socioeconomic status, background, ethnicity, as well as residence status may be a possible explanation for this discrepancy, as not all non-Japanese patients with infectious disease were traveling in our study. Another explanation may be that the country of origin for the immigrants to Japan may have improved their public health interventions, which was described about the United States in an analysis by the Centers for Disease Control and Prevention.^47^

Regarding specific outcomes demonstrating significant differences between non-Japanese ethnicities and Japanese patients, we found significantly more deaths from East Asian patients diagnosed with injury in comparison to Japanese patients. Specifically, we found that the majority of East Asian patients diagnosed with injury were due to car accidents (30%) and injuries while under the influence of alcohol (22%), with 18% of the car accidents from intoxicated patients. A study conducted by Arthur et al in the United States resulted with similar outcomes, where Asian patients were found to have higher mortality rates from injuries compared to Caucasian patients.^48^ In addition, Asian patients had a higher percentage of motor vehicle accidents compared to all other ethnicities observed in the study.^48^ We also observed significantly more overdose diagnoses in the South/Southeast Asian and Middle Eastern group compared to the Japanese group. Although the patients had overdosed on substances such as bleach, preservatives, and prescribed medications, none had consumed alcohol or opiates, which was inconsistent with studies reporting a recent global surge in these overdose deaths and hospitalizations.^49^^,^^50^ Considering this and the fact that there were only a small number of non-Japanese overdose cases, future studies on this topic are warranted. Finally, East Asian and South/Southeast Asian and Middle Eastern patients diagnosed with CPAOA also had a significantly longer length of stay compared to Japanese CPAOA patients. This may be due to the difference in age for CPAOA diagnoses (mean age for Japanese: 53.5, South/Southeast and Middle East: 53.7, and East Asian: 57.0), but more likely due to the difference in severity (mean GCS for Japanese: 3.8, South/Southeast and Middle East: 3.0, and East Asian: 3.0), as increased severity of illness is known to lead to longer hospitalizations in the ICU.^51^ Regardless, the aforementioned should be further investigated in future studies.

As host of the Olympic and Paralympic Games in 2021, and the 2025 World Exposition, it is paramount for Japan to prepare for the influx of non-Japanese residents and tourists, and to realize an appropriate non-Japanese patient care system that can be standardized throughout the nation. A preliminary step may be to consider nations with slightly higher international population ratios than Japan such as Finland,^10^ and observe their non-national patient care practices and their outcomes.

### Limitations

There are several limitations to this study. First, the data is limited to ED outpatient visits requiring tertiary care and secondary or tertiary care admissions in a single hospital: TMDU’s Trauma and Acute Critical Care Center during 2010–2019. Further comparisons and analysis of healthcare facilities throughout Japan with a larger sample size must be conducted to develop a holistic understanding for non-national patient care. Second, the categorization of patients by nationality is based on various indicators including name and clinical summaries, due to the current lack of a standardized method to inquire patients about their nationality and background (eg, residents or tourists in Japan). Factors such as the existence of patients with Japanese names who cannot speak Japanese or non-national patients who are fluent and understand Japanese culture have not been considered. Third, approximately half of all patient records from 2012 at the TMDU’s Trauma and Acute Critical Care Center are missing from the digital patient database due to a power outage at the hospital. The existence of missing data may have affected and distorted the results discussed in this study: however, we believed the inclusion of this would have generated more biased results than its removal. Fourth, although we took severity into account using GCS scores, we were unable to adjust for variables such as comorbidities. In addition to transfers, taking source or site information into account may have also been preferable, especially for infectious disease diagnoses. Since there are various methods to categorize severity other than GCS scores (eg, APACHE II scores^52^), the availability of data including underlying disease, comorbidities, or the presence of language barriers, may have enhanced the analysis, and thus future studies on this should be conducted. Fifth, our dataset did not include information regarding the patient’s type of health insurance or socio-economic background, which may have affected the patient’s choice of treatment or decision, admission, and length of stay. Although we did not find significant differences between non-Japanese and Japanese patient length of stay and outcome, further research regarding patient backgrounds may help shed light on various factors that affect patient choice. Sixth, the field of infectious disease consists of various diseases and disciplines. Future studies concerning specific infectious disease types are needed. Finally, as we were unable to differentiate non-Japanese tourists and residents (differences in the type of health insurance, socioeconomic status, or travel plans), as well as the arrival times (daytime or nighttime) this may have also affected outcomes and hospital lengths of stay.

### Conclusion

We observed more anaphylaxis, burn, and infectious disease diagnoses in non-Japanese patients, but no notable differences in mortality rates or lengths of stay regardless of severity in a tertiary emergency center in Japan. A societal redesign of residential, tourist, academic, and employment environments that ensure public health safety regardless of nationality, visa status, or language ability, may be pertinent, especially in the age of COVID-19.

## APPENDIX A. SUPPLEMENTARY DATA

eTable 1. Number and proportion of missing values for non-Japanese and Japanese patients at TMDU’s emergency department from 2010–2019 (except 2012)

eTable 2. Ethnicity breakdown and rationale behind categorization

eTable 3. Disease diagnosis breakdown (^*^Eleventh Edition of the Principal Diagnosis Short List for Emergency Medicine)

eTable 4. Length of stay stratified by age for patients diagnosed with cardiovascular disease, anaphylaxis, burns, or infectious disease

eTable 5. Percentage of diagnosis by ethnicity at TMDU’s emergency department from 2010–2019 (except 2012)

eTable 6. Mortality rates by ethnicity at TMDU’s emergency department from 2010–2019 (except 2012)

eTable 7. Length of stay by ethnicity at TMDU’s emergency department from 2010–2019 (except 2012)
