# Supplementary material for: Comparison of Disease Patterns and Outcomes Between Non-Japanese and Japanese Patients at a Single Tertiary Emergency Care Center in Japan
Source: J Epidemiol. 2022 Feb 5;32(2):80–8. doi: 10.2188/jea.JE20200211 (PMC8761568; doi:10.2188/jea.JE20200211)
Supplement: Supplementary file 1 [file je-32-080-s001.pdf]

eTable 1. Number and proportion of missing values for non-Japanese and Japanese patients at TMDU's emergency department from 2010–2019 (except 2012)

Covariates	Non-Japanese (N=373)<br>n (%)	Japanese (N=16,231)<br>n (%)
Sex	8 (2.1%)	233 (1.4%)
Age	1 (0.3%)	31 (0.2%)
Survival/Mortality	33 (8.8%)	1,368 (8.4%)
Visit or Admission/Discharge dates	37 (9.9%)	1,845 (11.4%)
GCS	16 (4.3%)	802 (4.9%)
Diagnosis	5 (1.3%)	231 (1.4%)

GCS, Glasgow Coma Scale.
Note: Some were duplicates

eTable 2. Ethnicity breakdown and rationale behind categorization

Ethnicity	Categorization	Examples[a]	n	%
East Asian	Chinese or Korean Names that are written in Katakana or Kanji, are included in the last name.	Chu, Chen	189	58.2
South Asian<br>South East Asian<br>Middle East	Names that are of Asian descent, but not East Asian	Nguyen, Huong, Patel, Singh	50	15.4
Caucasian<br>Hispanic	Western names for both first and last names and names with Spanish connotation	Robert Johnson, Phillip Smith, Carlos Sanchez	68	20.9
Unspecified	Non-Japanese names with Japanese last names (as this may be due to marriage)	Alexander Kawaguchi	18	5.5

[a] All example names do not reflect actual patient names

eTable 3. Disease diagnosis breakdown (*Eleventh Edition of the Principal Diagnosis Short List for Emergency Medicine)
CPA, cardiopulmonary arrest; CPAOA, cardiopulmonary arrest on arrival; ICD, International

Diagnosis	WHO ICD-11 Name
Injury	Injury, poisoning or certain other consequences of external causes: injuries
Gastrointestinal	Diseases of the digestive system (gastrointestinal; including liver, gallbladder disease)
Cerebrovascular	Diseases of the nervous system: cerebrovascular diseases
Cardiovascular	Diseases of the circulatory system
Overdose/Toxin	Injury, poisoning or certain other consequences of external causes: harmful effects of substances including poisons, toxins
CPAOA	Symptoms, signs or clinical findings, not elsewhere classified: cardiopulmonary arrest on arrival
Neurological	Diseases of the nervous system
Pulmonary	Diseases of the respiratory system
Infectious	Certain infectious or parasitic diseases
Endocrine	Endocrine, nutritional, or metabolic diseases
CPA	Symptoms, signs or clinical findings, not elsewhere classified: cardiopulmonary arrest at hospital/after arrival
Anaphylaxis	Diseases of the immune system: allergic or hypersensitivity conditions: anaphylaxis
Burns	Injury, poisoning or certain other consequences of external causes: burns
Self-Harm	External causes of morbidity or mortality: self-harm
Hemo-Immuno	Diseases of the blood or blood forming organs and diseases of the immune system
Psychiatry	Mental, behavioral or neurodevelopmental disorders
Genitourinary	Any disease characterized by pathological changes to the genitourinary system
Other	Unclassified

Classification of Diseases; WHO, World Health Organization;
* Incorporates the WHO’s International Classification of Diseases

eTable 4. Length of stay stratified by age for patients diagnosed with cardiovascular disease, anaphylaxis, burns, or infectious disease

			Percentage		Length of Stay	
			Non-Japanese n (%)	Japanese n (%)	Non-Japanese Median (IQR)	Japanese Median (IQR)
Cardiovascular		Total	14 (4.3%)	1,097 (8.2%)	3.5 (5.0)	3.0 (11.0)
	Age Range	0–14	0 (0.0%)	4 (0.03%)	- (-)	2.5 (5.5)
		15–64	10 (3.1%)	377 (2.8%)	3.5 (1.8)	4.0 (10.0)
		≥64	4 (1.2%)	715 (5.3%)	5.0 (6.5)	3.0 (12.0)
Anaphylaxis		Total	21 (6.5%)	176 (1.3%)	2.0 (0.0)	2.0 (1.0)
	Age Range	0–14	4 (1.2%)	10 (0.1%)	2.0 (0.3)	2.0 (1.0)
		15–64	17 (5.2%)	139 (1.0%)	2.0 (1.0)	2.0 (1.0)
		≥64	0 (0.0%)	27 (0.2%)	- (-)	2.0 (1.0)
Burns		Total	6 (1.8%)	76 (0.6%)	1.5 (1.0)	3.0 (9.3)
	Age Range	0–14	1 (0.3%)	9 (0.1%)	2.0 (0.0)	1.0 (8.0)
		15–64	5 (1.5%)	42 (0.3%)	1.0 (1.0)	2.0 (6.0)
		≥64	0 (0.0%)	25 (0.2%)	- (-)	8.0 (12.0)
Infectious		Total	17 (5.2%)	486 (3.6%)	4.0 (5.0)	7.0 (11.0)
	Age Range	0–14	1 (0.3%)	2 (0.01%)	1.0 (0.0)	7.5 (3.5)
		15–64	12 (3.7%)	158 (1.2%)	4.0 (5.8)	6.0 (13.8)
		≥64	4 (1.2%)	326 (2.4%)	4.0 (1.5)	7.0 (10.8)

IQR, interquartile range.
“-” denotes n= 0 or 1 for either the Non-Japanese or Japanese sample, and thus cannot be computed
TMDU’s emergency department from 2010–2019 (except 2012, N=13,695)

eTable 5. Percentage of diagnosis by ethnicity at TMDU's emergency department from 2010–2019 (except 2012)

Diagnosis	Percentage				
	Japanese (N=13,370)	Caucasian/Hispanic (N=68)	East Asian (N=189)	South/Southeast Asian/Middle Eastern (N=50)	Unspecified (N=18)
	n (%)	n (%)	n (%)	n (%)	n (%)
Injury	2,892 (21.6%)	15 (22.1%)	37 (19.6%)	8 (16.0%)	3 (16.7%)
Gastrointestinal	2,000 (15.0%)	7 (10.3%)	38 (20.1%)	10 (20.0%)	1 (5.6%)
Cerebrovascular	1,423 (10.6%)	9 (13.2%)	20 (10.6%)	6 (12.0%)	2 (11.1%)
Cardiovascular	1,097 (8.2%)	3 (4.4%)	10 (5.3%)	0 (0.0%)	1 (5.6%)
Overdose/ Toxin	965 (7.2%)	3 (4.4%)	12 (6.3%)	**8 (16.0%)**	**5 (27.8%)**
CPAOA	803 (6.0%)	5 (7.4%)	9 (4.8%)	3 (6.0%)	0 (0.0%)
Neurological	806 (6.0%)	8 (11.8%)	5 (2.6%)	0 (0.0%)	1 (5.6%)
Pulmonary	612 (4.6%)	1 (1.5%)	9 (4.8%)	4 (8.0%)	2 (11.1%)
Infectious	486 (3.6%)	4 (5.9%)	11 (5.8%)	2 (4.0%)	0 (0.0%)
Endocrine	286 (2.1%)	3 (4.4%)	2 (1.1%)	1 (2.0%)	0 (0.0%)
CPA	270 (2.0%)	1 (1.5%)	4 (2.1%)	0 (0.0%)	0 (0.0%)
Anaphylaxis	176 (1.3%)	**6 (8.8%)**	**12 (6.3%)**	1 (2.0%)	**2 (11.1%)**
Burns	76 (0.6%)	1 (1.5%)	2 (1.1%)	**3 (6.0%)**	0 (0.0%)
Self-Harm	66 (0.5%)	0 (0.0%)	0 (0.0%)	1 (2.0%)	1 (5.6%)
Hemo-Immuno	59 (0.4%)	0 (0.0%)	1 (0.5%)	0 (0.0%)	0 (0.0%)
Psychiatry	76 (0.6%)	0 (0.0%)	2 (1.1%)	0 (0.0%)	0 (0.0%)
Genitourinary	25 (0.2%)	0 (0.0%)	0 (0.0%)	0 (0.0%)	0 (0.0%)
Other	1,252 (9.4%)	2 (2.9%)	15 (7.9%)	3 (6.0%)	0 (0.0%)

CPA, cardiopulmonary arrest; CPAOA, cardiopulmonary arrest on arrival.

Analyzed using Chi-square

"-" denotes n= 0 or 1 for either the Non-Japanese or Japanese sample, and thus cannot be computed

* p-values < 0.05 are shown in bold

eTable 6. Mortality rates by ethnicity at TMDU's emergency department from 2010–2019 (except 2012)

Diagnosis	Mortality				
	Japanese n (%)	Caucasian /Hispanic n (%)	East Asian n (%)	South Asian/ Southeast Asian/ Middle Eastern n (%)	Unspecified n (%)
Total	1,539 (11.5%)	8 (11.8%)	21 (11.1%)	5 (10.0%)	0 (0.0%)
Injury	111 (0.8%)	1 (1.5%)	**5 (2.6%)**	1 (2.0%)	0 (0.0%)
Gastrointestinal	82 (0.6%)	0 (0.0%)	0 (0.0%)	0 (0.0%)	0 (0.0%)
Cerebrovascular	142 (1.1%)	1 (1.5%)	2 (1.1%)	1 (2.0%)	0 (0.0%)
Cardiovascular	96 (0.7%)	0 (0.0%)	0 (0.0%)	- (-)	0 (0.0%)
Overdose/Toxin	11 (0.1%)	0 (0.0%)	0 (0.0%)	0 (0.0%)	0 (0.0%)
CPAOA	740 (5.5%)	4 (5.9%)	6 (3.2%)	3 (6.0%)	- (-)
Neurological	11 (0.1%)	0 (0.0%)	0 (0.0%)	- (-)	0 (0.0%)
Pulmonary	57 (0.4%)	0 (0.0%)	1 (0.5%)	0 (0.0%)	0 (0.0%)
Infectious	52 (0.4%)	0 (0.0%)	1 (0.5%)	0 (0.0%)	- (-)
Endocrine	13 (0.1%)	0 (0.0%)	0 (0.0%)	0 (0.0%)	- (-)
CPA	101 (0.8%)	1 (1.5%)	0 (0.0%)	- (-)	- (-)
Anaphylaxis	3 (0.02%)	0 (0.0%)	0 (0.0%)	0 (0.0%)	0 (0.0%)
Burns	3 (0.02%)	0 (0.0%)	0 (0.0%)	0 (0.0%)	- (-)
Self-Harm	37 (0.3%)	- (-)	- (-)	0 (0.0%)	0 (0.0%)
Hemo-Immuno	5 (0.04%)	- (-)	0 (0.0%)	- (-)	- (-)
Psychiatry	0 (0.0%)	- (-)	0 (0.0%)	- (-)	- (-)
Genitourinary	0 (0.0%)	- (-)	- (-)	- (-)	- (-)
Other	75 (0.6%)	0 (0.0%)	0 (0.0%)	0 (0.0%)	- (-)

CPA, cardiopulmonary arrest; CPAOA, cardiopulmonary arrest on arrival.

"-" denotes n= 0 or 1 for either the Non-Japanese or Japanese sample, and thus cannot be computed

Analyzed with Fisher test

* p-values < 0.05 are shown in bold

eTable 7. Length of stay by ethnicity at TMDU's emergency department from 2010–2019 (except 2012)

Diagnosis	Length of Stay				
	Japanese Median (IQR)	Caucasian Hispanic Median (IQR)	East Asian Median (IQR)	South Asian Southeast Asian Middle Eastern Median (IQR)	Unspecified Median (IQR)
Total	3.0 (8.0)	2.0 (6.3)	3.0 (4.0)	3.0 (7.8)	2.0 (1.0)
Injury	3.0 (6.0)	3.0 (10.5)	2.0 (2.0)	2.0 (15.5)	3.0 (5.0)
Gastrointestinal	6.0 (8.0)	7.0 (8.0)	5.0 (5.8)	5.5 (4.8)	2.0 (0.0)
Cerebrovascular	6.0 (12.0)	5.0 (17.0)	4.0 (10.5)	4.5 (14.8)	1.0 (0.0)
Cardiovascular	3.0 (11.0)	3.0 (0.5)	4.5 (7.0)	- (-)	3.0 (0.0)
Overdose/Toxin	2.0 (2.0)	1.0 (0.5)	2.0 (1.0)	1.5 (1.0)	2.0 (1.0)
CPAOA	1.0 (0.0)	1.0 (5.0)	**2.0 (11.0)**	**2.0 (1.0)**	- (-)
Neurological	2.0 (3.0)	1.5 (1.0)	1.0 (1.0)	- (-)	1.0 (0.0)
Pulmonary	5.0 (8.0)	1.0 (0.0)	4.0 (4.0)	5.5 (7.5)	5.0 (2.0)
Infectious	7.0 (11.0)	3.0 (3.8)	4.0 (4.0)	2.0 (1.0)	- (-)
Endocrine	4.0 (9.0)	5.0 (1.0)	4.5 (3.5)	7.0 (0.0)	- (-)
CPA	4.0 (9.0)	2.0 (0.0)	4.0 (1.5)	- (-)	- (-)
Anaphylaxis	2.0 (1.0)	1.5 (1.0)	2.0 (0.0)	4.0 (0.0)	2.0 (0.0)
Burns	3.0 (9.3)	6.0 (0.0)	1.5 (0.5)	1.0 (0.5)	- (-)
Self-Harm	1.0 (1.0)	- (-)	- (-)	8.0 (0.0)	1.0 (0.0)
Hemo-Immuno	6.0 (9.5)	- (-)	2.0 (0.0)	- (-)	- (-)
Psychiatry	2.0 (3.3)	- (-)	1.0 (0.0)	- (-)	- (-)
Genitourinary	6.0 (12.0)	- (-)	- (-)	- (-)	- (-)
Other	3.0 (7.0)	3.0 (10.5)	**2.0 (2.0)**	2.0 (15.5)	- (-)

CPA, cardiopulmonary arrest; CPAOA, cardiopulmonary arrest on arrival; IQR, interquartile range; SD, standard deviation.

Analyzed with Mann-Whitney U Test

"-" denotes n= 0 or 1 for either the Non-Japanese or Japanese sample, and thus cannot be computed

[*] p-values<0.05 are shown in bold
